# Psychological Impact of Autism Screening on Caregivers

**DOI:** 10.1002/aur.70267

**Published:** 2026-04-30

**Authors:** Ramkumar Aishworiya, Devon Gangi, Van Kim Ma, Chandni Parikh, Nor Azyati Yusoff, Narueporn Likhitweerawong, Sally Ozonoff

**Affiliations:** ^1^ Khoo Teck Puat‐National University Children's Medical Institute National University Health System Singapore; ^2^ Department of Pediatrics, Yong Loo Lin School of Medicine National University of Singapore Singapore; ^3^ Department of Psychiatry, MIND Institute University of California, Davis Sacramento California USA; ^4^ Department of Pediatrics University of California, Davis Sacramento California USA; ^5^ Rady Children's Hospital Autism Discovery Institute San Diego California USA; ^6^ Department of Pediatrics, Faculty of Medicine and Health Sciences Universiti Putra Malaysia Serdang Selangor Malaysia; ^7^ Department of Pediatrics Chiang Mai University Chiang Mai Thailand

**Keywords:** autism, caregiver, harms, psychological impact, screening

## Abstract

Autism screening in childhood is common, yet little is known about its potential psychological impact on caregivers. The U.S. Preventive Services Task Force, an independent national panel of disease prevention experts, stated that this gap in knowledge limited their ability to endorse universal autism screening. This study examined the psychological impact of autism screening, using data from a large community‐based sample (*n* = 1272) involving online caregiver‐completed autism screeners at child age 6, 9, 12, 18, and 24 months. Caregivers completed the Participation Impact Questionnaire retrospectively (mean child age at completion 37.2 ± 4.8 months) to measure feelings about screening. A minority (34.7%) of the sample reported presence of ≥ 1 negative feeling; the most commonly endorsed was “worried”. Among this subset, negative feelings were of short duration (lasted for < 1 day in 56.9%), were mild in severity (86.4%), and did not affect functioning (85.3%). A majority (86.2%) also reported ≥ 1 positive feeling. Our findings address a critical evidence gap regarding potential harms of autism screening and support universal screening, given that psychological harms are not common and have low functional impact, as well as possible psychological benefits.

## Lay Summary

Although screening for presence of autism among young children is commonly done, little is known about its potential psychological impact on caregivers. There is very limited information related to this topic currently, with the U.S. Preventive Services Task Force, an independent national panel of experts, stating that this gap in knowledge limited their ability to make recommendations related to guidelines on routine autism screening for all children. This study addresses this knowledge gap and aimed to study the potential psychological impact (harms, as well as benefits) of autism screening in caregivers. Data was obtained from a longitudinal study involving online caregiver‐completed autism screeners at child age 6, 9, 12, 18, and 24 months. Caregivers were notified at child age 18–24 months if their children had screened positive at any time‐point and offered diagnostic evaluation to determine a final outcome by 36 months. Subsequently caregivers were invited to complete the Participation Impact Questionnaire (PIQ) which was designed to measure negative and positive feelings about screening, their duration, and functional impact. In our study, a total of 1272 caregivers completed the PIQ. Only a minority (34.7%) reported presence of ≥ 1 negative feeling; the most commonly endorsed feeling was “worried”. Among those who reported these feeling(s), they were of short duration, were mild in severity and did not affect day‐to‐day activities in the majority. A majority (86.2%) also reported ≥ 1 positive feelings; the most common ones were “interested” and “curious.” Study findings suggest that psychological harms reported by caregivers following autism screening are less common than positive feelings and even if present, have short duration, low functional impact, and low severity. Our findings are in support of recommendations toward universal autism screening recommendations, given low harm potential and possible psychological benefits of screening on caregivers.

## 1 Introduction

There has been increasing emphasis on the importance of early identification in autism to ensure timely access to behavioral interventions during crucial stages of brain development and plasticity. Previous studies have demonstrated that autism can be reliably diagnosed as early as 18 months (Pierce et al. 2019), allowing for earlier access to interventions which is associated with better developmental progress, including improved communication and social skills (Guthrie et al. 2023). In tandem, significant research has examined the validity of screening instruments for autism, mostly suggesting acceptable sensitivity of instruments like the Modified Checklist for Autism in Toddlers, Revised, with Follow‐Up and thereby supporting routine use for daily clinical practice (Aishworiya et al. 2023; Levy et al. 2020). Indeed, the American Academy of Pediatrics recommends routine screening for autism at 18 and 24 months for all children (Hyman et al. 2020).

The benefits of autism screening have been generally well articulated. Proponents cite the critical role of systematic screening in facilitating an autism diagnosis and thereby subsequent treatment initiation at younger ages (Wieckowski et al. 2021; Zwaigenbaum et al. 2019). Studies have demonstrated that children identified via systematic screening are referred for autism evaluation at a younger age than those referred following parental or physician concerns (Vivanti et al. 2025). Furthermore, in many settings, screening results can help families access resources related to early intervention and advocate for specific therapies. Recent studies find that a significant proportion (48%–54%) of pediatric primary care practices conduct systematic screening for autism as part of routine practice (Carbone et al. 2020; Guthrie et al. 2019). In contrast, there is much less scientific evidence regarding the potential harms of autism screening on children and caregivers. In its 2016 landmark paper, the U.S. Preventive Services Task Force (USPSTF) noted that no study presented data relevant to harms of autism screening in young children (Levy et al. 2020; McPheeters et al. 2016; Siu et al. 2016).

Research on screening for other conditions such as cancer has suggested several direct and indirect effects from the screening process. These include adverse psychological feelings related to anticipatory anxiety of the screening result and subsequent anticipation of a possible diagnosis, unnecessary emotional distress after a false positive screening, and lack of support following diagnosis (Brewer et al. 2007; Byrne et al. 2008; DeFrank et al. 2015). Specific to autism screening, two qualitative studies exploring potential harms were recently published. One examined perspectives of pediatricians (*n* = 10), early intervention providers (*n* = 51), and parents (*n* = 22) to generate a taxonomy pertinent to harms of autism screening (Petruccelli et al. 2022). Some of the harms identified in this study were loss of a “typically developing” identity of the child, social withdrawal, parent–child relational strain, and time and cost burdens. Respondents from all three groups reported feelings of psychological distress such as anxiety and worry related to the screening process, but the frequency and duration of these feelings was not measured, given the qualitative methodology. Another recent small qualitative study focused on parents (*n* = 26) whose child received a false‐positive autism screening (Eilenberg et al. 2022). Parents reported experiencing emotional distress while waiting for diagnostic results but also reported benefits (increased knowledge of autism, corroboration of existing concerns, connection to services) which they felt outweighed the emotional costs.

These limited studies highlight the need for further research on the potential harms of autism screening and factors that may be associated with perceived harm, including demographics of caregivers and results of screening. This evidence gap is critical to address, to allow informed guidelines on universal autism screening; this is addressed in the present study. Utilizing a large dataset of families who participated in an autism screening study, we aimed to understand the psychological impact of autism screening among caregivers. Given the limited existing literature related to this topic, the study's hypothesis was exploratory in focusing on the possible range and nature of the psychological effects that caregivers experienced.

## 2 Methods

### 2.1 Overview of Procedures

This study utilized data from a longitudinal investigation testing three screeners for autism in a general community sample of 2005 children. All methods were approved by the university's Institutional Review Board in accordance with ethical standards. Members of the autistic community were not specifically involved in this study design. Following informed consent, caregivers completed three online screening measures at 6, 9, 12, 18, and 24 months.

Study screening status was defined dichotomously: screen positives (SPs) had scores at or over the cutoff on at least one instrument at least once between 6 and 24 months, while screen negatives (SNs) were below autism spectrum disorder (ASD) cutoffs on all three instruments at all ages. Given the number of screeners administered at multiple ages and the large sample size, it was not feasible to provide individualized screening results on each instrument at each age, so parents were informed only if their child had screened positive on any instrument at any age. Parents were notified of SP status when their child was 18–24 months and offered a tele‐diagnostic assessment at both 24 and 36 months. 52.6% of children were in the SP group and 47.4% were in the SN group.

Parents were told at the beginning of participation that there were two parts to the study. All participants would complete the first part of the study, the online screenings from 6 to 24 months. A subset of participants, whose children screened positive (on any screener at any age), would be invited for the second part of the study (diagnostic tele‐assessments). To preserve clinician blinding regarding screening status, approximately 10% of SN children were randomly selected to also complete diagnostic evaluations. Parents were informed that if their child was not above screening cutoffs or randomized to get an assessment, they would not be invited to the second part of the study. Parents of children in the SN group did not receive any further notification of study screening status. Parents of children in the SP group were notified of their child's study screening status via email, using the following script: “As you may recall from our initial intake, this study tests different online screening methods of identifying developmental delays, including delayed speech and autism spectrum disorder, in the community. It is a standard part of our study protocol to invite families for an evaluation if a child scores over a cutoff on any of the screeners at any age to determine how well the online screeners correspond with behavioral observations. In many cases, the evaluation will determine that the child may not actually have any concerns. Either way, you will be given some developmental feedback and recommendations, if applicable.”

At the conclusion of the study, when all participants were ≥ 36 months, the PIQ was sent to parents.

### 2.2 Participants

Participants were recruited from the community via hospital records, state birth records, and social media. Families were eligible to participate if they had: (a) a child under the age of 10 months, (b) a valid email address, (c) access to a device with internet capacity, and (d) the ability to complete questionnaires at a fifth grade English reading level. Participants were not excluded based on any genetic or medical conditions, family history, or prematurity; 99.4% of the sample were enrolled by and screened at 6 months (mean age of enrolment = 5.77 months, SD = 2.06).

### 2.3 Measures

#### 2.3.1 Online Screening Questionnaires (6–24 Months)

##### 2.3.1.1 Modified‐Checklist for Autism in Toddlers, Revised (M‐CHAT‐R)

This is a parent‐report checklist with 20 yes/no items normed for use with toddlers aged 16–30 months (Robins et al. 2014) with pooled sensitivity of 83% (95% CI: 77%–88%) and specificity of 94% (95% CI: 89%–97%) through meta‐analysis (Wieckowski et al. 2023). The M‐CHAT‐R was completed at child ages 18 and 24 months. A positive screen was defined as ≥ 3 (Robins et al. 2014).

##### 2.3.1.2 Infant‐Toddler Checklist (ITC)

This parent‐report questionnaire is normed for ages 6–24 months and measures social‐communication, language, and symbolic development (Wetherby et al. 2008). Caregivers completed the ITC at 6, 9, 12, 18, and 24 months. Scores ≤ 10th percentile on any subscale are defined as SPs (Wetherby et al. 2008).

##### 2.3.1.3 Video‐Referenced Infant Rating System for Autism (VIRSA)

This video‐based screening tool presents pairs of videos of parents and infants playing together (Young et al. 2020). Videos depict a range of social‐communication ability, with scores ranging from 1 (least social) to 10 (most social). The tool requires forced‐choice judgments in which parents select the video that is most like how their child usually behaves. The VIRSA has satisfactory test–retest reliability and convergent validity and differentiates autism from non‐autism in elevated familial likelihood samples as early as 12 months (Young et al. 2020). Caregivers completed the VIRSA when their child was 6, 9, 12, 18, and 24 months; a positive screen on the VIRSA was a score ≤ 3.

#### 2.3.2 Outcome Measures

##### 2.3.2.1 PIQ

During the final months of study funding, all participants who were still enrolled in the study (*n* = 1977 of 2005) were contacted and asked to fill out a PIQ, developed for this study to understand the impact of completing autism screeners. The PIQ was administered retrospectively after all screening questionnaires and study visits had been completed (mean age at completion 37.2 months, SD = 4.8). Therefore, when completing the PIQ, caregivers were aware both of their child's overall study screening status (SP/SN) and the outcome of diagnostic assessments.

The PIQ asked up to seven questions about the process of completing the online screeners. Caregivers were first asked if the items in the screening questionnaires “captured any concerns [they] already had” or caused them “to have any new concerns” about their child's development or behavior. Participants were next asked to select if they experienced any negative feelings while completing the screening questionnaires (choosing as many as were relevant from: *worried, upset, confused, surprised, angry, unsettled, disagreed, don't remember*). If a caregiver indicated they had experienced negative feelings, they were asked how severe the feelings were (*barely noticeable, mild, moderate, intense*, or *don't remember*), how long they lasted (*less than a day, more than a day but less than a week, more than a week but less than a month, a month or more*, or *don't remember*), and how much they affected daily functioning or ability to carry out important tasks (*not at all, just a little, a moderate amount, a lot*, or *don't remember*). Caregivers were also asked if they experienced any positive feelings while completing the screeners (choosing as many as they wished from: *curious, relieved, informed, reassured, interested, grateful, supported, don't remember*).

##### 2.3.2.2 Outcome Classification

At 36 months, all children were categorized into outcome groups. Participants who were both screened online and seen for a tele‐assessment were classified into one of two outcome groups based on the diagnostic evaluation. The ASD group (*n* = 65) included participants who met *DSM‐5* ASD criteria and had obtained a score at or over the autism cutoff of 12 on the TELE‐ASD‐PEDS (TAP), a telehealth measure used to assess autism in toddlers in their own homes via two‐way video conferencing (Wagner et al. 2025). The Non‐ASD group (*n* = 597) did not meet DSM‐5 criteria for ASD based on the tele‐assessment. Participants who were not seen for a diagnostic assessment (*n* = 610) were classified into the Screening Only group.

#### 2.3.3 Analysis Plan

Data analysis was conducted using SPSS version 25 (IBM Corp 2017). First, we measured caregivers' reactions to completing autism screeners, calculating the rates of negative and positive emotions, their duration, and functional impact. Then we examined whether responses differed based on study screening status (SP vs. SN), outcome classification (ASD, Non‐ASD, screening only), and demographic variables (i.e., child sex, race, ethnicity, parental education, household income, child birth order) using *χ* ^2^ tests.

## 3 Results

The final sample comprised 1272 caregivers who completed the PIQ (from a total of 1977 sent the form, 64.3% response rate). Demographic information for the full PIQ sample is presented in Table 1. Respondents were more likely to have higher household income, higher maternal and paternal education, a child in the SP group, and a child in the Non‐ASD group (than the screening only group) compared to nonrespondents. There were no differences in other child/family demographic variables (Table S1) between those in the full study who did not complete the PIQ and those included in the present analyses who did complete the PIQ.

**TABLE 1:** Descriptive and demographic data of sample.

	ASD (*N* = 65), *n* (%)	Non‐ASD (*N* = 597), *n* (%)	Screening Only (*N* = 610), *n* (%)
*Child characteristics*			
Sex			
Male	47 (72.3%)	341 (57.1%)	299 (49.0%)
Female	18 (27.7%)	256 (42.9%)	311 (51.0%)
Race			
Black or African American	6 (9.2%)	11 (1.8%)	15 (2.5%)
Asian	11 (16.9%)	64 (10.7%)	44 (7.2%)
American Indian or Alaskan Native	0 (0.0%)	4 (0.7%)	8 (1.3%)
Native Hawaiian or Pacific Islander	0 (0.0%)	3 (0.5%)	0 (0.0%)
White	20 (30.8%)	349 (58.5%)	371 (60.8%)
Multiple races	27 (41.5%)	161 (27.0%)	166 (27.2%)
Not reported	1 (1.5%)	5 (0.8%)	6 (1.0%)
Ethnicity			
Hispanic/Latino	20 (30.8%)	145 (24.3%)	146 (23.9%)
Not Hispanic/Latino	43 (66.2%)	443 (74.2%)	458 (75.1%)
Not reported	2 (3.1%)	9 (1.5%)	6 (1.0%)
Birth order			
First born	30 (46.2%)	264 (44.2%)	305 (50.0%)
Later born	35 (53.8%)	333 (55.8%)	304 (49.8%)
Study screening status			
Screen positive	65 (100%)	560 (93.8%)	44 (7.2%)
Screen negative	0 (0.0%)	37 (6.2%)	566 (92.8%)
*Caregiver and family characteristics*			
Maternal race			
Black or African American	6 (9.2%)	11 (1.8%)	17 (2.8%)
Asian	11 (16.9%)	103 (17.3%)	64 (10.5%)
American Indian or Alaskan Native	0 (0.0%)	4 (0.7%)	6 (1.0%)
Native Hawaiian or Pacific Islander	1 (1.5%)	4 (0.7%)	2 (0.3%)
White	30 (46.2%)	378 (63.3%)	413 (67.7%)
Multiple races	12 (18.5%)	61 (10.2%)	70 (11.5%)
Not reported	5 (7.7%)	36 (6.0%)	38 (6.2%)
Maternal education			
Less than college	19 (29.2%)	66 (11.1%)	85 (13.9%)
College and higher	46 (70.8%)	531 (88.9%)	524 (85.9%)
Not reported	0 (0.0%)	0 (0.0%)	1 (0.2%)
Paternal race			
Black or African American	6 (9.2%)	18 (3.0%)	35 (5.7%)
Asian	10 (15.4%)	69 (11.6%)	61 (10.0%)
American Indian or Alaskan Native	1 (1.5%)	4 (0.7%)	6 (1.0%)
Native Hawaiian or Pacific Islander	0 (0.0%)	9 (1.5%)	1 (0.2%)
White	34 (52.3%)	391 (65.5%)	407 (66.7%)
Multiple races	9 (13.8%)	14 (2.3%)	52 (8.5%)
Not reported	5 (7.7%)	52 (8.7%)	48 (7.9%)
Paternal education			
Less than college	26 (40.0%)	144 (24.1%)	174 (28.5%)
College and higher	39 (60.0%)	453 (75.9%)	435 (71.3%)
Not reported	0 (0.0%)	0 (0.0%)	1 (0.2%)
Annual household income			
< $100k	32 (49.2%)	186 (31.2%)	207 (33.9%)
> $100k	28 (43.1%)	364 (61.0%)	366 (60.0%)
Not reported	5 (7.7%)	47 (7.9%)	37 (6.1%)

**FIGURE 1:**
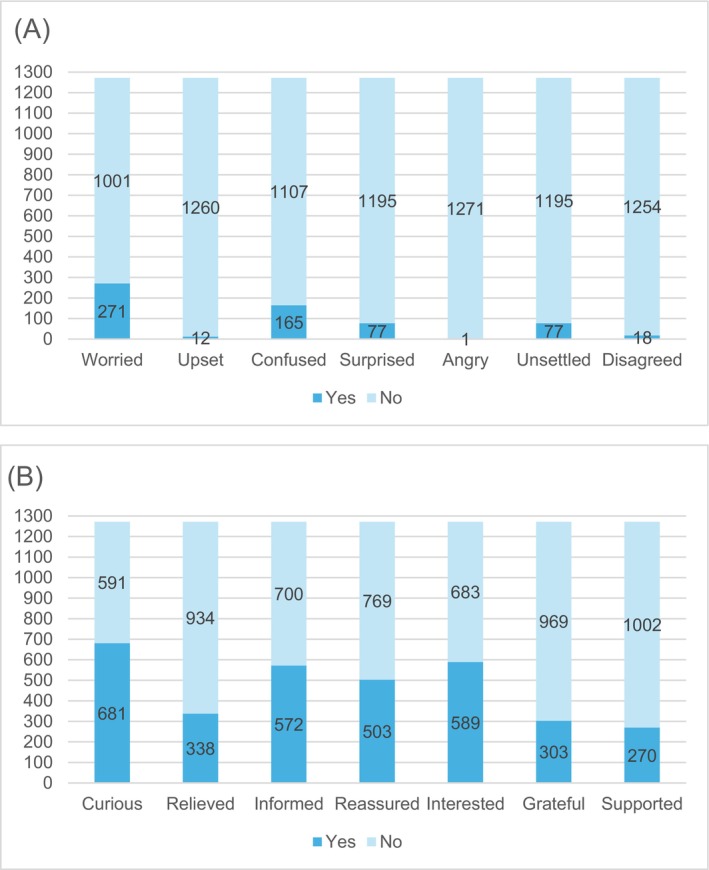
Distribution of negative feelings (A) and positive feelings (B) endorsed by caregivers after completing screening questionnaires.

Most caregivers (84.7%) reported that screening did not raise new concerns related to their child's development. Screening captured pre‐existing concerns in 21.5% of respondents, while 43.0% did not have any pre‐existing concerns. A substantial minority of respondents (*n* = 441, 34.7%) reported having at least 1 negative feeling while completing the screeners. The most frequently reported negative feeling was “worried” (21.3%), followed by “confused” (13.0%) (see Figure 1). However, among those who reported negative feelings, the majority reported that these feelings did not persist (56.9% reported “less than a day”), did not affect functioning (85.3% reported “not at all”), and were not severe (87.5% reported “barely noticeable” or “mild”) (Table 2). Negative feelings were more likely in respondents whose child had screened positive compared to those who screened negative, *χ* ^2^(1) = 23.5, *p* < 0.001 (see Table 3). Such feelings were also more frequent in caregivers in the ASD outcome group than the Non‐ASD or Screening Only groups, *χ* ^2^(2) = 50.58, *p* < 0.001 (see Table 3). Parents whose children were first‐born were more likely to report negative feelings than those with later‐born children (42.6% vs. 27.7%), *χ* ^2^(1) = 31.0, *p* < 0.001. Families with maternal education of a college degree or higher were more likely to report negative feelings than those without a college degree (36.8% vs. 26.2%), *χ* ^2^(1) = 9.80, *p* = 0.002. The proportion of respondents who endorsed negative feelings did not differ based on child sex, race, or ethnicity, paternal education, or household income, *p*s > 0.06.

**TABLE 2:** Positive and negative feelings experienced while completing screening questionnaires.

Variables	Yes, *n* (%)	No, *n* (%)
Did screening items capture pre‐existing concerns^a^	274 (21.5%)	397 (31.2%)
Did screening items cause new concerns^b^	167 (13.1%)	1077 (84.7%)
At least 1 positive feeling endorsed	1097 (86.2%)	175 (13.8%)
At least 1 negative feeling endorsed	441 (34.7%)	831 (65.3%)
*Severity of negative feelings*		
Barely noticeable	116 (26.3%)	NA
Mild	265 (60.1%)	NA
Moderate	55 (12.5%)	NA
Intense	0 (0.0%)	NA
I don't remember	5 (1.1%)	NA
*Duration of negative feelings*		
Less than a day	251 (56.9%)	NA
More than a day but less than a week	108 (24.5%)	NA
More than a week but less than a month	24 (5.4%)	NA
A month or more	32 (7.3%)	NA
I don't remember	26 (5.9%)	NA
*Impact of negative feelings on daily functioning*		
Not at all	376 (85.3%)	NA
Just a little	47 (10.7%)	NA
A moderate amount	11 (2.5%)	NA
A lot	4 (0.9%)	NA
I don't remember	3 (0.7%)	NA

^a^ Does not include 601 participants who indicated they did not have any pre‐existing concerns.

^b^ Does not include 28 participants who endorsed “I don't remember.”

**TABLE 3:** Frequencies of participants experiencing positive and negative feelings by group.

Group	Positive feelings endorsed (*N* = 1097)			Negative feelings endorsed (*N* = 441)		
	*n* (%)	*χ* ^2^	*p*	*n* (%)	*χ* ^2^	*p*
*Outcome classification*						
ASD	56 (86%)	1.41	0.494	46 (71%)	50.58	< 0.001
Non‐ASD	522 (87%)			223 (37%)		
Screening Only	519 (85%)			172 (28%)		
*Study screening status*						
Screen positive	579 (87%)	0.11	0.739	273 (41%)	23.47	< 0.001
Screen negative	518 (86%)			168 (28%)		

Positive feelings were commonly experienced by caregivers, with 1097 respondents (86.2%) endorsing at least 1 positive feeling while completing screening questionnaires (Table 2). The most frequently reported positive feeling was “curious” (53.5%), followed by “interested” (46.3%) and “informed” (45.0%) (Figure 1). Positive feelings were significantly more likely in respondents whose children were first‐born than later‐born (90.3% vs. 82.6%), *χ* ^2^(1) = 15.9, *p* < 0.001. Families with maternal education of a college degree or higher were more likely to report positive feelings than those without a college degree (87.3% vs. 81.9%), *χ* ^2^(1) = 4.97, *p* = 0.026. There were no significant differences in the proportion of respondents who endorsed positive feelings based on study screening status, outcome group, child sex, race, or ethnicity, paternal education, or household income, *p*s > 0.21.

## 4 Discussion

For many years, there was a void in knowledge regarding potential psychological harms of autism screening, as noted by the USPSTF (Siu et al. 2016). Recent qualitative studies catalogued possible harms and benefits, but samples were small and did not address how frequently such feelings occurred, duration, or functional impact. Our quantitative study addressed this scientific gap by examining the psychological impact of autism screening in a large community‐based sample of parents who participated in screenings when their child was 6–24 months. We found that approximately a third of the sample reported negative feelings, but these feelings were mild and short‐lived. Nonetheless, this emphasizes the need to address these feelings and provide appropriate support for these caregivers. Additionally, we found that most respondents also endorsed positive feelings, suggesting potential psychological benefits of autism screening.

Specifically, 34.7% of caregivers reported 1 or more negative feelings, with “worried” being the most common. This supports previous findings that parents mainly feel anxious and worried (Petruccelli et al. 2022). Yet, it is reassuring that in our sample, even among those who reported negative feelings, they were not severe, long‐lasting, or affecting their functioning. This corroborates another previous study's finding that even parents who reported harms related to false‐positive screens did not have long‐term negative impacts on their well‐being (Eilenberg et al. 2022). Overall, our results from this large community‐based sample add weight to the conclusion that psychological harms of autism screening are not substantial, thereby supporting recommendations for universal autism screening.

We found that negative psychological feelings were more likely in those whose child had a positive screening result as well as in those with an eventual ASD diagnosis. Due to the timing and retrospective nature of the PIQ completion in our study, caregivers' negative feelings may have been affected by awareness of their child's screening results and diagnostic outcome. Nonetheless, these findings emphasize the critical role of assessing for psychological harm and including psychological support as needed as part of the screening process for autism. In the presence of resource limitations, this may be offered especially to first‐time parents and to caregivers whose children receive a positive screening result and might range from online resources to in‐person counseling support.

The majority of participants also reported positive feelings, which is consistent with the qualitative findings from Eilenberg et al. (2022). Although we were not able to elucidate the specific reasons behind these emotions, feeling “reassured” and “relieved” could suggest that screening provides an affirmation for parents regarding their child's development. Interestingly, many parents reported feeling “curious” and “interested” while completing the screeners. This may indicate that some parents would welcome further specific information about child development and/or autism, which could potentially be incorporated into the screening process.

Strengths of this study include being the first quantitative examination of the psychological impact of autism screening on parents, measuring not only the rate of negative feelings but also their duration and impact on parental functioning. The large, racially diverse sample underwent repeated screenings from infancy to toddlerhood. Screenings were completed online, with diagnostic evaluations offered to participants as part of the research study, at no cost to families, after positive results. Our results should be interpreted within the context of a longitudinal research study; further research is still needed to examine the rates of negative and positive feelings among recipients of routine screening in primary care.

There are also several limitations. First, the PIQ was completed at the end of the study, after completion of all screening timepoints, and may not have captured the true feelings at the point of screening or may have been influenced by knowledge of the child's screening status and eventual diagnostic results. Future studies should administer questionnaires about psychological effects concurrently with screenings. Second, our respondents were skewed toward higher education levels, limiting generalizability of study findings. Finally, the PIQ did not measure as comprehensive a list of possible psychological harms as recently catalogued by others (Eilenberg et al. 2022; Petruccelli et al. 2022).

## 5 Conclusion

This study found a relatively low prevalence of negative feelings experienced by caregivers after autism screening, which had a short duration and little long‐term functional impact. Positive feelings were far more common (86%) than negative feelings (35%). Our findings help address the crucial evidence gap identified by the USPSTF (Siu et al. 2016) regarding potential harms of autism screening and support recommendations for universal autism screening (Hyman et al. 2020), given that screening benefits outweighed possible harms in this study. Nonetheless, our findings suggest a need to consider the psychological impact on parents when designing routine clinical autism screening services and provide targeted support, especially for those who may be at higher likelihood of experiencing negative feelings, such as those whose children have a screening result of concern.

## Author Contributions

Ramkumar Aishworiya assisted in study conceptualization, had full access to all data, conducted data analyses and interpretation, had responsibility for integrity of the data and accuracy of the analyses, wrote the initial manuscript, and critically reviewed and revised the manuscript. Devon Gangi assisted in study conceptualization and design, acquired and contributed data, had full access to the data, conducted data analysis and interpretation, and critically reviewed and revised versions of the manuscript. Van Kim Ma, Nor Azyati Yusoff, and Narueporn Likhitweerawong participated in study conceptualization and data interpretation, and critically reviewed and revised the manuscript. Chandni Parikh assisted in study conceptualization and design, acquired and contributed data, and critically reviewed and revised versions of the manuscript. Sally Ozonoff conceptualized and designed the study, acquired funding, contributed data, assisted in the interpretation of data, and critically reviewed and revised the manuscript. All authors approved the final manuscript as submitted and agree to be accountable for all aspects of the work.

## Funding

This work was supported by a grant from the National Institutes of Health, R01MH121344 (Ozonoff). The content is solely the responsibility of the authors and does not necessarily represent the official views of the NIH.

## Ethics Statement

All methods were approved by the university's Institutional Review Board in accordance with ethical standards (IRB Number 1164035‐16).

## Consent

Informed consent was obtained prior to caregivers completing the online screening measures.

## Conflicts of Interest

S.O. reports travel reimbursements and honoraria from Autism Speaks and Autism Science Foundation and book royalties from Guilford Press. The other authors declare no conflicts of interest.

## Supporting information


**Table S1:** Characteristics of PIQ respondents and nonrespondents.

## Data Availability

De‐identified individual participant data will not be made available due to IRB regulations. Data from the longitudinal study is available in the National Database for Autism Research (NDAR).
