# Supplementary material for: Psychological Impact of Autism Screening on Caregivers
Source: Autism Res. 2026 Apr 30;19(6):e70267. doi: 10.1002/aur.70267 (PMC13174677; doi:10.1002/aur.70267)
Supplement: Supplementary file 1 — Table S1: Characteristics of PIQ respondents and nonrespondents. [file AUR-19-0-s001.docx]

**Supplemental Table 1. Characteristics of PIQ respondents and non-respondents**

|  | **Respondents** | **Non-Respondents** |
| --- | --- | --- |
| **Child Characteristics** | ***n* (%)** | ***n* (%)** |
| *Sex* |  |  |
| Male  Female | 687 (54.0%)  585 (46.0%) | 379 (53.8%)  326 (46.2%) |
| *Race* |  |  |
| Black or African American | 32 (2.5%) | 19 (2.7%) |
| Asian | 119 (9.4%) | 57 (8.1%) |
| American Indian or Alaskan Native | 12 (0.9%) | 6 (0.9%) |
| Native Hawaiian or Pacific Islander | 3 (0.2%) | 4 (0.6%) |
| White | 740 (58.2%) | 406 (57.6%) |
| Multiple Races | 354 (27.8%) | 208 (29.5%) |
| Not Reported | 12 (0.9%) | 5 (0.7%) |
| *Ethnicity* |  |  |
| Hispanic/Latino | 311 (24.4%) | 184 (26.1%) |
| Not Hispanic/Latino  Unknown | 44 (3.5%)  17 (1.3%) | 514 (72.9%)  7 (1.0%) |
| *Birth Order* |  |  |
| First Born | 599 (47.1%) | 355 (50.4%) |
| Later Born | 672 (52.8%) | 345 (48.9%) |
| *Study Screening Status** |  |  |
| Screen Positive | 669 (52.6%) | 299 (42.4%) |
| Screen Negative | 603 (47.4%) | 405 (57.4%) |
| *Outcome Group* |  |  |
| ASD | 65 (5.1%) | 31 (4.4%) |
| Non-ASD | 597 (46.9%) | 198 (28.1%) |
| Screening Only | 610 (48.0%) | 476 (67.5%) |
| **Caregiver and Family Characteristics** |  |  |
| *Maternal Race*  Black or African American  Asian  American Indian or Alaskan Native  Native Hawaiian or Pacific Islander  White  Multiple Races  Not Reported | 34 (2.7%)  178 (14.0%)  10 (0.8%)  7 (0.6%)  821 (64.5%)  143 (11.2%)  79 (6.2%) | 26 (3.7%)  84 (11.9%)  4 (0.6%)  3 (0.4%)  449 (63.7%)  89 (12.6%)  50 (7.1%) |
| *Maternal Education**  Less than college  College and higher | 248 (19.5%)  1023 (80.4%) | 208 (29.5%)  492 (69.8%) |
| Not Reported | 1 (0.1%) | 5 (0.7%) |
| *Paternal Race*  Black or African American  Asian  American Indian or Alaskan Native  Native Hawaiian or Pacific Islander  White  Multiple Races  Not Reported | 59 (4.6%)  140 (11.0%)  11 (0.9%)  10 (0.8%)  832 (65.4%)  115 (9.0%)  105 (8.3%) | 29 (4.1%)  69 (9.8%)  5 (0.7%)  4 (0.6%)  433 (61.4%)  85 (12.1%)  80 (11.3%) |
| *Paternal Education**  Less than college  College and higher | 426 (33.5%)  845 (66.4%) | 268 (38.0%)  432 (61.3%) |
| Not Reported | 1 (0.1%) | 5 (0.7%) |
| *Annual Household Income**  <$100,000  >$100,000 | 425 (33.4%)  757 (59.5%) | 288 (40.9%)  360 (51.1%) |
| Not Reported | 90 (7.1%) | 57 (8.1%) |

Note. * indicates significant difference between groups, *p*<.05
